# Are Behçet’s disease patients with Budd–Chiari syndrome at increased risk for the development of pulmonary hypertension?

**DOI:** 10.1093/rheumatology/keae067

**Published:** 2024-02-01

**Authors:** Mustafa Ekici, Serez İleri, Erdinç Ünaldı, Gözde Sevgi Kart Bayram, Levent Kılıç, Ali Akdoğan

**Affiliations:** Department of Rheumatology, Hacettepe University Faculty of Medicine, Altındağ, Ankara, Turkey; Department of Internal Medicine, Hacettepe University Faculty of Medicine, Altındağ, Ankara, Turkey; Department of Rheumatology, Hacettepe University Faculty of Medicine, Altındağ, Ankara, Turkey; Department of Rheumatology, Hacettepe University Faculty of Medicine, Altındağ, Ankara, Turkey; Department of Rheumatology, Hacettepe University Faculty of Medicine, Altındağ, Ankara, Turkey; Department of Rheumatology, Hacettepe University Faculty of Medicine, Altındağ, Ankara, Turkey

Rheumatology key message

- Including transthoracic echocardiogram in the workup of Behçet's disease with Budd-Chiari syndrome at presentation and follow-up seems reasonable.

Dear Editor, Budd–Chiari syndrome (BCS) is a severe type of vascular involvement in Behçet’s disease with a prevalence of 2.4–14.4% [1, 2]. Portal hypertension is a well-known cause of pulmonary arterial hypertension (PAH). PAH due to portal hypertension has a poor prognosis; survival of these patients is less than 50% at 5 years [3]. Behçet’s disease patients with BCS may be at increased risk for the development of PAH with their vulnerable endothelium [4]. In this study, we retrospectively investigated the frequency of pulmonary hypertension (PH) in Behçet’s patients with BCS.

Twenty-five Behçet’s patients with BCS among 902 patients from the Behçet’s disease database of Hacettepe University, Rheumatology Department were included in the study. Patients’ data including demographic and clinical features, were obtained retrospectively from hospital records. All BD patients with BCS fulfilled the International Study Group for Behçet's disease criteria [5]. The first and last transthoracic echocardiogram (TTE) results of the Behçet’s patients and serum haemoglobin levels at the times of TTE evaluations were also noted. PH was considered if estimated systolic arterial pressure was ≥40 mmHg by TTE.

The frequency of BCS in Behçet's patients was 25/902 (2.77%). Nineteen (76%) patients were male, and the mean age was 43.8 (12) years. The median follow-up time for Behçet’s disease was 13.9 (range 9.6–22.1) years and for BCS was 5.5 (range 2.4–12.1) years. In three patients the diagnosis of BCS was made before the diagnosis of Behçet’s disease, and in three patients the diagnosis of BCS and Behçet’s disease were made simultaneously. The median time to BCS in patients with a previous diagnosis of Behçet’s disease was 7.6 (range 14) years. One patient (6.25%) developed cirrhosis after BCS. Sixteen (64%) out of 25 patients had at least one TTE evaluation after the diagnosis of BCS. Among 25 patients, nine (36%) patients had dyspnoea at initial presentation. Dyspnoea was present in seven (43.8%) of those who underwent TTE evaluation and in two (22.2%) of those who did not (*P* = 0.4). Patient characteristics of those evaluated by TTE are shown in Table 1. The median estimated sPAP of the BCS patients was 30 mmHg (range 25–35). The sPAP of nine (56.25%) patients was reported to be ≥30 mmHg. One patient had an estimated sPAP of 40 mmHg and one other had low ejection fraction. Only six patients had a control TTE, in whom the median sPAP was 25 mmHg (range 25–31.3). The median values of haemoglobin measured simultaneously at the first and last TTE were 13.4 (±2.4) and 12.6 (±2.1) mg/dl, respectively. Two patients (12.5%) died during follow-up, but the exact cause of death could not be determined.

**Table 1.** Clinical characteristics of Behçet's patients with Budd–Chiari syndrome

Patient	Age, years, gender	Behçet's disease duration, years	Budd–Chiari duration, years	Vascular involvement	Comorbidity	**Symptoms^a^**	First sPAP/EF	Last sPAP/EF
1	49, M	15.5	15.2	VCI, RCCA, RCIA	Ø	Dyspnoea	40 mmHg/64%	—
2^b^	63, M	32.1	0.5	VCI, VCS, PAI	COPD	Dyspnoea orthopnoea, PND	35 mmHg/30%	35 mmHg/30%
3^b^	77, M	30.6	0.2	VCI	HT, COPD, CRF	Dyspnoea, fatigue	35 mmHg/60%	—
4	41, M	18,8	6.2	VCI, DVT	Ø	Ø	35 mmHg/63%	—
5	42, F	23.1	25.1	VCI + HV, PAI	Cirrhosis	Ø	35 mmHg/60%	—
6	26, M	3.2	4.4	VCI + HV, PAI	Ø	Ø	35 mmHg/65%	25 mmHg/69%
7	46, M	22.1	4.0	HV, DVT, PAI		Dyspnoea	30 mmHg/65%	30 mmHg/60%
8	41, M	7.7	0.1	VCI, DVT	Ø	Ø	30 mmHg/62%	—
9	43, F	11.1	11.1	HV, PAI		Ø	30 mmHg/65%	—
10	22, M	10.4	5.3	VCI + HV, REIA, RCFA, SSS, RTS	Ø	Ø	25 mmHg/60%	—
11	42, F	24.1	5.5	VCI + HV	HT, hypothyroidism	Ø	25 mmHg/68%	—
12	42, M	5.3	5.0	VCI	Ø	Ø	25 mmHg/65%	—
13	38, M	14.1	7.8	VCI + HV	Ø	Dyspnoea	25 mmHg/60%	—
14	49, M	8.8	4.7	VCI + HV	HT, DM, CAD	Dyspnoea	20 mmHg/50%	25 mmHg/50%
15	33, F	13.1	5.6	VCI, DVT	Ø	Dyspnoea	20 mmHg/68%	25 mmHg/65%
16	43, M	22.2	13.8	VCI, LAA	Ø	Ø	20 mmHg/60%	25 mmHg/60%

a Symptom: findings before the first transthoracic echocardiogram.

b Exitus. Ø: absent. CAD: Coronary artery disease; COPD: chronic obstructive pulmonary disease; CRF: chronic renal failure; DM: diabetes mellitus; DVT: deep vein thrombosis; EF: ejection fraction; HT: hypertension; HV: hepatic vein; LAA: left axillary artery; PAI: pulmonary arterial involvement; PND: paroxysmal nocturnal dyspnoea; RCCA: right common carotid artery; RCFA: right common femoral artery; RCIA: right common iliac artery; REIA: right external iliac artery; RTS: right transverse sinus; sPAP: systolic pulmonary artery pressure; SSS: superior sagittal sinüs; VCI: vena cava inferior; VCS: vena cava superior.

In this study, although many of the patients with BCS had mild elevations in estimated sPAP by TTE at presentation or during follow-up, there were no patients with severe PH leading to right heart failure. Sudden cardiac death is a complication of severe PH [6]. In our study two patients died during follow-up, for whom we could not determine the exact cause of death. Although hepatic failure is considered to be the major cause of mortality in Behçet’s patients with BCS, these two patients had PH but did not have advanced liver disease [2]. On the other hand they had serious comorbidities such as left heart failure or chronic renal failure.

It is hard to determine the exact cause of PH in systemic diseases such as Behçet’s disease. Severe group IV PH due to pulmonary arterial involvement (PAI) that needs to be treated by endarterectomy or PAH specific agents can occur in Behçet’s disease [7, 8]. Moreover these patients are at increased risk for the development of PH due to cardiac involvement or drugs that are used frequently to control the disease activity such as IFN-α or cyclophosphamide [8]. As expected, there were five patients who had both BCS and PAI in our study [1]. The presence of more than one clinical condition associated with PH development is not rare in systemic diseases. Portal hypertension due to BCS may contribute to the development of severe PH in this patient group by leading to an increased exposure of pulmonary circulation to vasoactive substances [9].

Our study has some limitations. This was a retrospective study, and none of the patients were evaluated by right heart catheterization, which is the gold standard for PH diagnosis. To define the presence of PH, we used only sPAP values and did not present data about the right ventricle.

A significant number of patients with Behçet’s disease and BCS had dyspnoea, though there were none with severe PH in our study. It seems reasonable to include TTE in the workup of Behçet’s disease patients with BCS at presentation and at least follow them up clinically for the signs of PH both for therapeutic decision making and increasing data about the topic.

## Data Availability

Data are available upon reasonable request by any qualified researchers who engage in rigorous, independent scientific research, and will be provided following review and approval of a research proposal and Statistical Analysis Plan (SAP) and execution of a Data Sharing Agreement (DSA). All data relevant to the study are included in the article.
